# Integrated Phenotypic and Transcriptomic Analysis of the Effects of a Novel Arylaminoalcohol on *Plasmodium falciparum*

**DOI:** 10.3390/biology15201837

**Published:** 2026-10-14

**Authors:** Yaël Pronnier Lefebvre, Ibtissem Ben Gayed, Aurélien Aubry, Patrice Agnamey, Romain Mustière, Alexandra Dassonville-Klimpt, Pascal Sonnet, Yohann Le Govic, Anne Totet, Céline Damiani

**Affiliations:** 1 Service de Parasitologie et Mycologie Médicales, Centre de Biologie Humaine, CHU Amiens-Picardie,1 Rond-Point du Pr Cabrol, 80054 Amiens, France; yaelpronlef@msn.com (Y.P.L.); bengayed.ibtissem@chu-amiens.fr (I.B.G.); legovic.yohann@chu-amiens.fr (Y.L.G.); totet.anne@chu-amiens.fr (A.T.); 2 Agents Infectieux, Résistance et Chimiothérapie (AGIR), UR 4294, Université de Picardie Jules Verne, Chemin du Thil, 80025 Amiens, France; aubry.aurelien@chu-amiens.fr (A.A.); p.agnamey@u-picardie.fr (P.A.); romain.mustiere@u-picardie.fr (R.M.); alexandra.dassonville@u-picardie.fr (A.D.-K.); pascal.sonnet@u-picardie.fr (P.S.); 3 Service de Virologie, Centre de Biologie Humaine, CHU Amiens-Picardie,1 Rond-Point du Pr Cabrol, 80054 Amiens, France

**Keywords:** *Plasmodium falciparum*, antimalarial compounds, arylaminoalcohols, mechanism of action, microscopy, transcriptomics, gene ontology, RNA-Seq

## Abstract

Malaria remains the leading parasitic infection worldwide, and the effectiveness of antimalarial drugs is increasingly threatened by the emergence of multidrug-resistant *Plasmodium falciparum* (*P. falciparum*) strains. In this context, our team previously developed a series of novel aminopyridinemethanol derivatives, among which GBPS336 showed potent nanomolar activity against *P. falciparum* strains with reduced susceptibility to reference antimalarials. Here, we investigated the effects of GBPS336 on the intraerythrocytic developmental cycle and transcriptional response of the parasite by combining microscopic phenotyping with whole-transcriptome RNA sequencing (Illumina) of synchronized *P. falciparum* 3D7 cultures exposed or not to the compound. In GBPS336-treated cultures, microscopic analysis revealed reduced parasitaemia, a marked delay and disruption of intraerythrocytic development and morphological alterations indicative of a parasite stress response. Transcriptomic profiling identified 356 differentially expressed genes at H24 post-exposition in ring-stage cultures with enrichment in functional categories associated with erythrocyte invasion and the parasite–host interface. Altogether, these findings demonstrate the antimalarial activity of GBPS336 and highlight invasion-related pathways as a potentially important component of its mechanism of action. Further studies will be required to determine whether these pathways are directly affected and to identify the molecular target(s) underlying the activity of the compound.

## Simple Summary

Malaria is a vector-borne disease caused by apicomplexan parasites that invade and multiply inside human red blood cells. It is responsible for hundreds of thousands of deaths every year, mostly in young children from sub-Saharan Africa. The effectiveness of antimalarial drugs is threatened by the emergence of resistant parasites. In this context, new compounds with novel biological properties are urgently needed. Our research team has designed a family of new arylaminoalcohols, and one of them, called GBPS336, shows potent antimalarial activity at very low doses, including parasites that already resist current treatments. In this study, we investigated the effects of GBPS336 on parasite development by combining microscopic examination with transcriptomic analysis. Under microscopy, the treated parasites showed a delayed maturation, became far less numerous, and appeared damaged. At the gene level, the compound mainly disturbed genes involved in red blood cell entry. These results suggest that GBPS336 interferes with the parasite’s ability to invade host cells but they do not by themselves demonstrate a direct effect on invasion or ascertain the compound’s molecular target.

## 1. Introduction

Malaria remains one of the world’s most important infectious diseases, with an estimated 282 million cases and 610,000 deaths reported in 2024, the majority of which were attributable to *P. falciparum* infection [1]. The high generalization of artemisinin-based combination therapies (ACTs) for uncomplicated malaria led to a reduction in mortality and morbidity during the past two decades. However, the development of resistance to artemisinins and their partner drugs is threatening the gains made in the recent years in the battle against malaria [1,2,3]. Consequently, the identification and characterization of novel antimalarial compounds remain major priorities for global health.

The use of high-throughput screening techniques has contributed significantly to the pipeline of antimalarial drug discovery over the past decade by facilitating the identification of compounds with strong antiparasitic activity regardless of the molecular target [4,5]. These techniques have sped up the process of lead compounds’ identification. However, elucidating the mechanisms through which candidate molecules exert their biological activity remains a major challenge. Understanding parasite responses to drug exposure is essential not only for optimizing lead compounds but also for predicting resistance mechanisms [5,6].

Technological developments in transcriptomics have transformed the study of interactions between host and pathogen and between drugs and parasites by providing a systemic view of how cells respond to chemical stimuli [6,7]. These techniques do not directly identify the molecular target of a compound but contribute to a better understanding of the biological processes and regulatory networks involved when parasites adapt to stress. Studies on *P. falciparum* have shown that transcriptional responses are triggered by both environmental and chemical stresses [8,9,10].

Arylaminoalcohols constitute an important and clinically validated class of antimalarial drugs, including quinine, mefloquine and lumefantrine. While the mode of action of some members of this class, such as quinine, has been relatively well characterized in relation to the parasite’s haem metabolism, the molecular basis of mefloquine activity remains controversial, with several mechanisms having been proposed. Suggested mechanisms of action include ribosomal inhibition and interference with haem detoxification, while resistance-associated adaptations continue to emerge [11,12,13]. GBPS336 is a novel synthetic arylaminoalcohol derivative belonging to a series of aminopyridinemethanol compounds previously developed by our team [14,15,16,17]. It has demonstrated potent in vitro activity against *P. falciparum* W2 and 3D7 strains [18]. GBPS336 also demonstrated in vivo efficacy comparable to mefloquine in a mouse model of *Plasmodium berghei* ANKA infection at a dose of 10 mg/kg (unpublished data). However, the biological response induced by GBPS336 has not yet been investigated at the molecular level.

In this study, we combined synchronized parasite cultures, microscopic phenotypic assessment and whole-transcriptome analysis to characterize the response of *P. falciparum* to GBPS336 over a 48 h exposure period. By integrating phenotypic observations with transcriptomic data, we aimed to relate the morphological and developmental alterations induced by drug exposure to the biological processes and pathways affected at the transcriptional level, thereby gaining further insight into the parasite response to GBPS336.

## 2. Materials and Methods

### 2.1. Plasmodium Falciparum Culture and Synchronization

The chloroquine-sensitive *P. falciparum* 3D7 (*Pf*3D7) reference strain, a well-characterized laboratory strain widely used in antimalarial drug-response studies, was maintained in continuous in vitro culture according to the method described by Trager and Jensen [19]. Parasites were cultured at 37 °C in a humidified atmosphere with 5% CO_2_ using RPMI 1640 medium (Sigma-Aldrich, Lyon, France) supplemented with Albumax^®^ (Life Technologies, Paisley, UK), hypoxanthine (Sigma-Aldrich), gentamicin (Sigma-Aldrich), and washed human erythrocytes obtained from healthy volunteers. Cultures were maintained in a total volume of 30 mL at a final haematocrit of 3%. Culture medium was renewed daily, whereas erythrocytes were replaced every 48 h. Routine parasitaemia was monitored by microscopic examination (full staining protocol detailed in Section 2.3).

To obtain highly synchronized parasite populations, cultures exhibiting a parasitaemia of approximately 5% were treated with 5% D-sorbitol (Sigma-Aldrich) according to the protocol described by Lambros and Vanderberg [20]. Synchronization efficiency was assessed microscopically and cultures were considered suitable for further experiments when more than 70% of infected erythrocytes contained parasites at the same developmental stage.

Two synchronized parasite populations were generated for the study: ring stage (43 h post-synchronization) and trophozoite stage (66 h post-synchronization); these corresponded to the developmental stages selected for drug exposure and subsequent phenotypic and transcriptomic analyses.

### 2.2. Experimental Design and Drug Exposure

Synchronized ring- and trophozoite-stage parasite cultures were exposed to GBPS336 at a concentration corresponding to twice the 50% inhibitory concentration (2xIC_50_). The IC_50_ value of GBPS336 against *Pf*3D7 was estimated at 9.73 ± 4.68 nM. in dose–response experiments previously conducted by our team [18], which corresponds to a concentration of approximately 19.5 nM. Untreated cultures were maintained under identical conditions and served as negative controls. Drug exposure was initiated immediately after the stages were selected by synchronization in order to ensure homogeneous parasite populations at the beginning of each experiment. At the time of drug exposure (H0), the mean parasitaemia was 5.1% in ring-stage cultures and 4.6% in trophozoite-stage cultures.

For each developmental stage, treated and untreated cultures were established in parallel from the same synchronized parasite population. Samples from the parasite cultures were collected at three predefined time points corresponding to baseline before treatment (H0) and 24 h (H24) and 48 h (H48) after drug exposure. At each sampling point, aliquots were collected for both microscopic examination and RNA extraction, allowing the integration of phenotypic observations with transcriptomic analyses.

Each experimental condition was performed using three independent biological replicates generated from separate parasite cultures established over three consecutive weeks. For each developmental stage (ring or trophozoite) and each replicate, five conditions were sampled—baseline (H0) and treated/untreated cultures at H24 and H48—yielding a total of 30 samples that were processed for microscopic analysis and RNA-seq.

### 2.3. Microscopic Phenotypic Assessment

The phenotypic effects of GBPS336 on *P. falciparum* development were assessed by microscopic examination of thin blood smears collected from each experimental condition at H0, H24 and H48 following drug exposure. Blood smears were air-dried, fixed with absolute methanol and stained with May-Grünwald-Giemsa. Slides were examined under oil immersion using a ×100 objective.

The experiments were performed using three independent biological replicates, and each smear was evaluated individually by four observers. The four observations were averaged for each biological replicate prior to statistical analysis.

Three quantitative microscopic parameters were recorded for every sample: parasitaemia, parasite developmental stage distribution and the proportion of pyknotic parasites. Mean values were subsequently calculated for each parameter prior to statistical analysis.

- Parasitaemia was determined by counting approximately 10,000 erythrocytes distributed across multiple microscopic fields and expressed as the percentage of infected erythrocytes relative to the total number of erythrocytes examined.
- Parasite developmental stages were identified according to standard morphological criteria and classified as ring, trophozoite or schizont stage parasites. The proportion of each developmental stage was expressed as a percentage of the total number of parasites counted.
- Pyknotic parasites were identified by the presence of condensed, intensely stained nuclear material associated with marked cytoplasmic degeneration and loss of normal parasite morphology. The proportion of pyknotic parasites was expressed as the percentage of pyknotic forms relative to the total number of erythrocytes examined. In addition, qualitative morphological features, including cytoplasmic vacuolization, abnormal parasite maturation and degenerative forms, were systematically documented over the 48 h exposure period.

Statistical analyses were performed using GraphPad Prism 10^®^ software (GraphPad Software Inc., Dotmatics, San Diego, CA, USA). Data on parasitaemia were compared using Student’s *t*-test. The distribution of parasitic stages between untreated parasites and those treated with GBPS336 was compared using Fisher’s exact test. Statistical comparisons were based on three independent biological replicates, for which the mean was calculated from the readings of four observers. A *p*-value < 0.05 was considered statistically significant.

### 2.4. RNA Extraction and Transcriptome Sequencing

Total RNA was extracted from the 30 samples previously preserved in TRIzol™ Reagent (Invitrogen™, Thermo Fisher Scientific, Wilmington, DE, USA) using the RNeasy Mini Kit^®^ (Qiagen, Hilden, Germany) on the automated QIAcube^®^ platform (Qiagen), according to the manufacturer’s instructions. RNA concentration and purity were assessed by spectrophotometry using a NanoDrop 2000^®^ instrument (Thermo Fisher Scientific, Wilmington, DE, USA).

RNA sequencing libraries were prepared from each RNA extract using the Illumina Stranded mRNA Prep^®^ kit (Illumina, San Diego, CA, USA) according to the manufacturer’s recommendations. Briefly, polyadenylated messenger RNAs were enriched using oligo(dT)-coupled magnetic beads, fragmented and reverse-transcribed into first-strand cDNA. Strand specificity was preserved during second-strand synthesis through incorporation of deoxyuridine triphosphates (dUTP). Sequencing adapters containing unique dual indexes were ligated to each library, followed by PCR amplification and purification using AMPure XP^®^ magnetic beads (Beckman Coulter, Brea, CA, USA).

Library concentration was quantified using a Qubit 4^®^ Fluorometer (Invitrogen™, Thermo Fisher Scientific). Individual libraries were normalized, pooled at equimolar concentrations to generate a multiplexed sequencing library and loaded onto a NextSeq 550^®^ sequencing system (Illumina). Paired-end sequencing was performed using a 60 Gb High Output flow cell (Illumina,) with 2 × 75 bp read chemistry (150 cycles), according to the manufacturer’s standard sequencing workflow.

### 2.5. Bioinformatic Analysis of RNA Sequencing Data

RNA-sequencing data were processed using a standardized bioinformatic workflow implemented on the Galaxy Europe platform (https://usegalaxy.eu, accessed on 7 October 2026) to generate gene expression profiles, identify differentially expressed genes (DEGs) and characterize the biological processes associated with GBPS336 exposure.

Raw sequencing reads were first trimmed to remove sequencing adapters and low-quality bases using Cutadapt (https://usegalaxy.eu, accessed on 7 October 2026), and read quality was assessed with FastQC (https://usegalaxy.eu, accessed on 7 October 2026). High-quality reads were subsequently aligned to the *P. falciparum* 3D7 reference genome (PlasmoDB) using the RNA STAR aligner (https://usegalaxy.eu, accessed on 7 October 2026).

Differential gene expression analyses were performed using DESeq2 by comparing GBPS336-treated parasites with untreated controls at each sampling time. Gene expression changes were expressed as log_2_ fold change (log_2_FC). Differentially expressed genes were defined using an adjusted *p*-value < 0.05 and an absolute log_2_FC greater than 1. Before performing analysis of drug response, 2452 genes that exhibited significantly different expression levels between the ring stage and trophozoite stage at H0 were removed to minimize the effect of the life cycle of the parasite on the experiment.

Global transcriptional variation among samples was explored by principal component analysis (PCA) generated within the Galaxy Europe environment (https://usegalaxy.eu, accessed on 7 October 2026). Differential expression profiles were further visualized using heatmaps generated with R software (version 4.4.3).

To facilitate the biological interpretation of transcriptomic alterations, functional enrichment analysis was performed using GOSeq (https://usegalaxy.eu, accessed on 7 October 2026) to identify significantly overrepresented Gene Ontology (GO) biological processes among differentially expressed genes.

## 3. Results

### 3.1. GBPS336 Induces Rapid Growth Inhibition and Morphological Alterations During the Intraerythrocytic Cycle

- In both synchronization conditions, GBPS336 exposure resulted in a significant reduction in parasitaemia compared with untreated controls. In ring-stage-synchronized cultures, parasitaemia at H24 was 71% of the baseline H0 value in GBPS336-treated cultures, compared with 96% in untreated controls (*p* < 0.05). At H48, parasitaemia decreased to 31% of the H0 value in treated cultures, compared with 89% in controls (*p* < 0.05). These values represent residual parasitaemia relative to baseline and should not be interpreted as parasite clearance rates. Similarly, in trophozoite-stage-synchronized cultures, parasitaemia at H24 was 73% of the initial H0 value in GBPS336-treated cultures, compared with 110% in controls (*p* < 0.05), and decreased to 22% versus 64%, respectively, at H48 (*p* < 0.05) (Figure 1).
- Microscopic examination revealed progressive morphological alterations throughout parasite development. In ring-stage cultures, treated parasites exhibited persistent ring forms associated with progressive cytoplasmic vacuolization (Figure 2). At later developmental stages, trophozoites showed impaired maturation (Figure 3), whereas schizonts displayed abnormal segmentation and marked structural disorganization (Figure 4).

**Figure 1:**
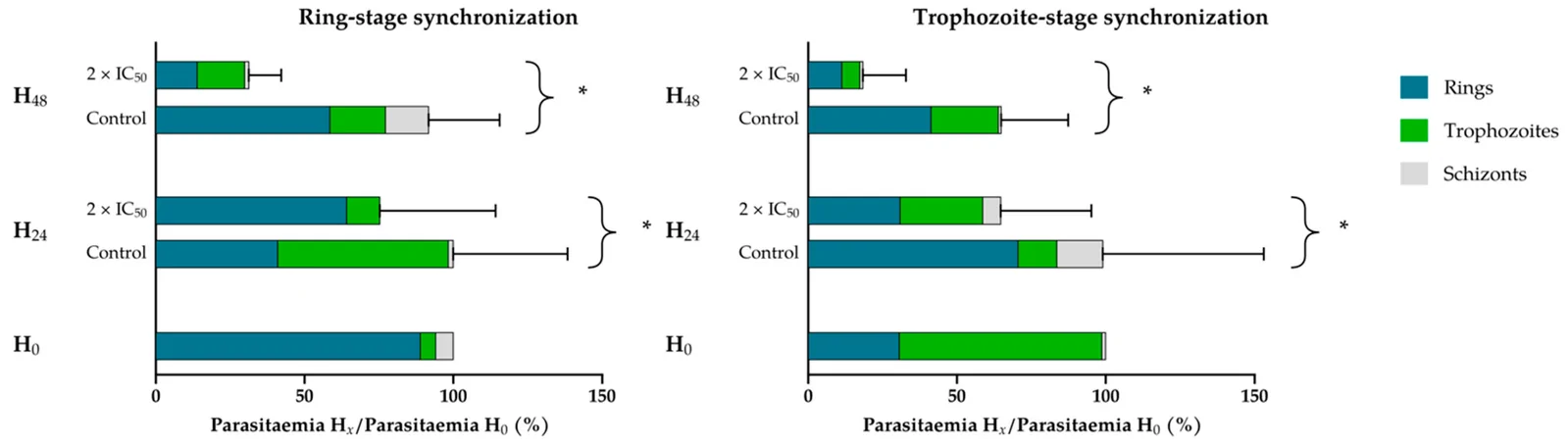
Distribution of parasite stages and changes in parasitaemia in *P. falciparum* cultures synchronized at the ring stage (**left** panel) and at the trophozoite stage (**right** panel) in the presence or absence of GBPS336. Parasitaemia at H24 and H48 is expressed as a ratio to the initial parasitaemia at H0 (* Student’s *t*-test, *p* < 0.05). Error bars represent standard deviation.

**Figure 2:**
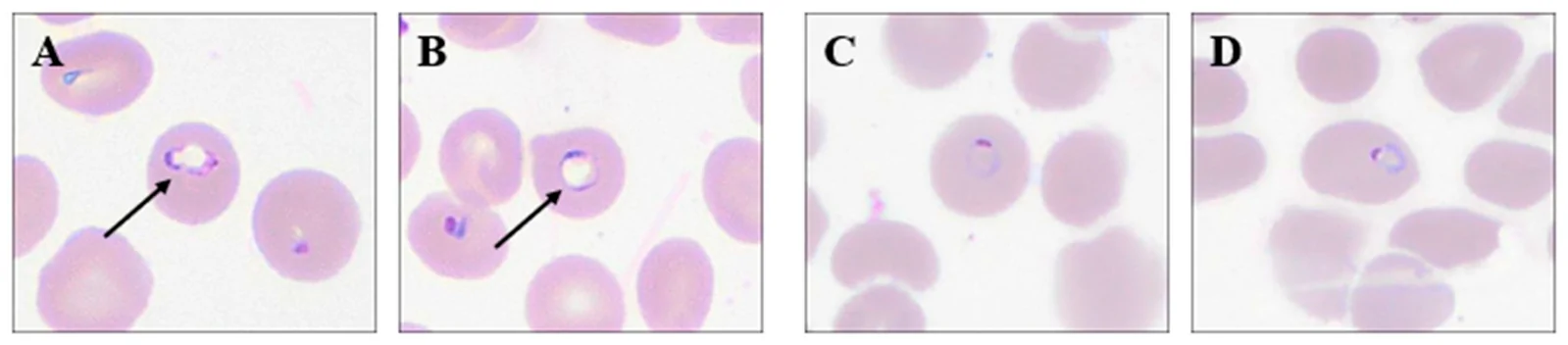
Morphological alterations of ring-stage parasites (thin blood smears, May–Grünwald–Giemsa staining, ×1000). (**A**,**B**) Rings exhibiting cytoplasmic vacuolization (arrows) from GBPS336-treated cultures; (**C**,**D**) rings with normal morphology from control cultures.

**Figure 3:**
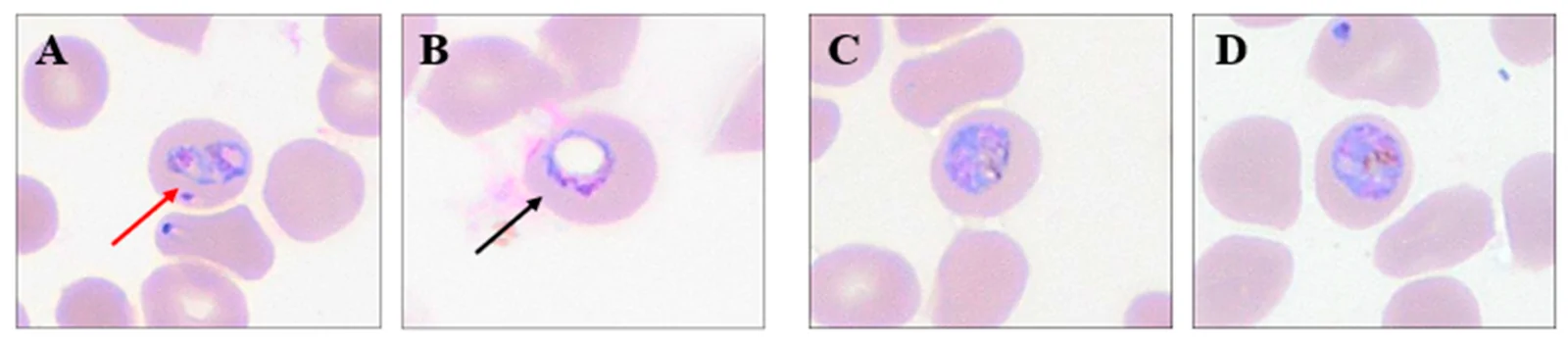
Morphological alterations of trophozoites (thin blood smears, May–Grünwald–Giemsa staining, ×1000). (**A**) Trophozoite with fragmented cytoplasm (red arrow) and (**B**) trophozoite showing cytoplasmic vacuolization (black arrow), both from GBPS336-treated cultures; (**C**,**D**) trophozoites with normal morphology from control cultures.

**Figure 4:**
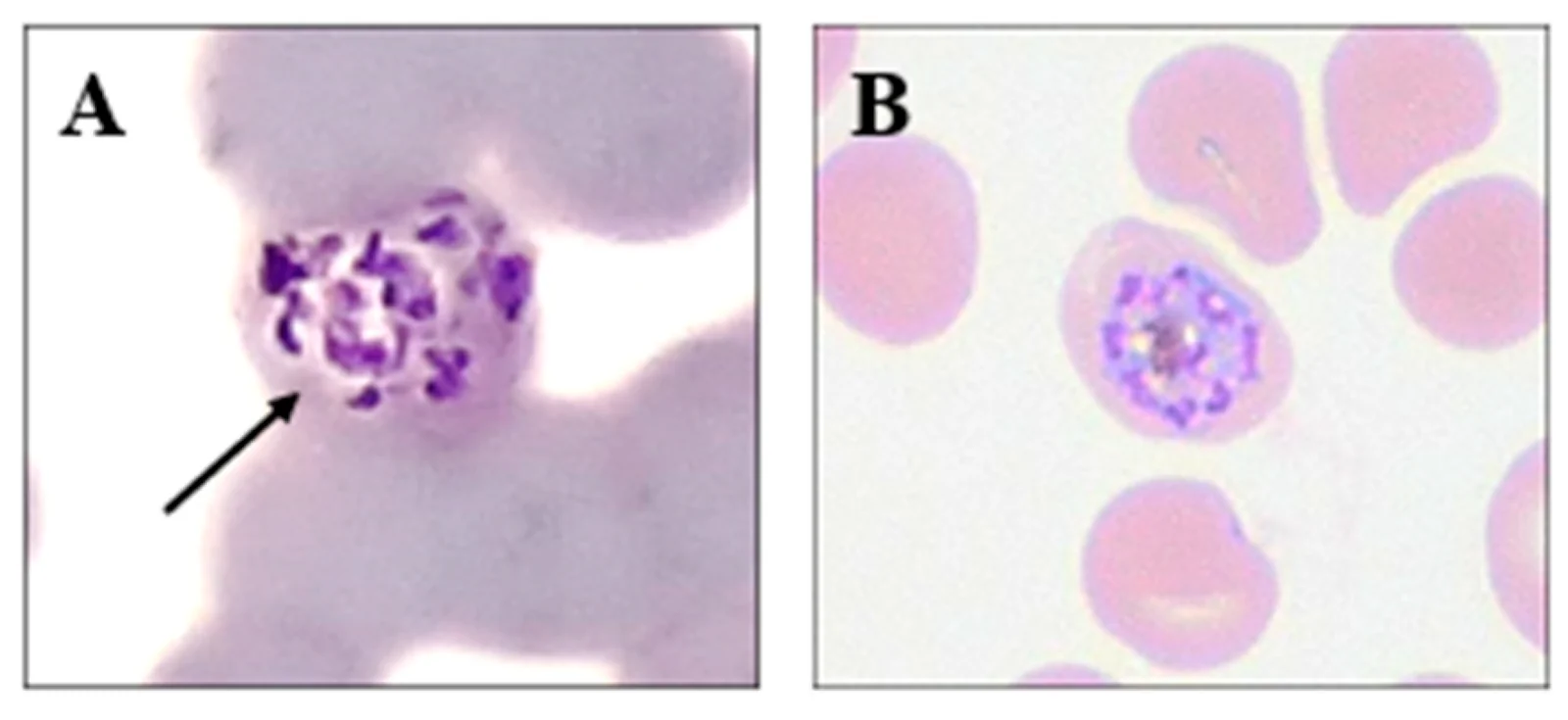
Morphological alterations of schizonts (thin blood smears, May–Grünwald–Giemsa staining, ×1000). (**A**) Schizont exhibiting a loss of merozoite arrangement (arrow) from a GBPS336-treated culture and (**B**) schizont with normal morphology from a control culture.

Overall, GBPS336 treatment was associated with reduced parasitic growth, progressive morphological alterations and increased pyknosis (Figure 5) in both synchronized cultures. These phenotypic abnormalities were observable as early as the first 24 h of treatment and persisted over the 48 h exposure period. Pyknotic forms accounted for 0.81% and 1.84% of the total erythrocytes examined at H24 and H48, respectively, in ring-stage synchronized cultures. In trophozoite-stage synchronized cultures, pyknotic forms represented 0.69% and 1.47% of the total erythrocytes examined at H24 and H48, respectively.

### 3.2. GBPS336 Alters the Transcriptional Trajectory of the Intraerythrocytic Developmental Cycle

- Among the 30 sequenced libraries, two (trophozoite stage, week 1, H0 and H24 treated with GBPS336) yielded fewer than 1 million reads and were excluded from downstream analysis. The remaining samples generated between 2 and 35 million reads and were retained for differential expression analysis. Transcriptomic profiles obtained from treated cultures were compared with those of untreated controls at the corresponding time points.
- Principal component analysis (PCA) reflected the continuous progression of the parasite through the intraerythrocytic cycle (Figure 6):
  ○ In untreated cultures, ring-stage transcriptomes at H24 clustered close to trophozoite-stage transcriptomes at H0, and ring-stage transcriptomes at H48 clustered close to trophozoite-stage transcriptomes at H24, consistent with normal developmental progression.
  ○ In contrast, GBPS336-treated ring-stage parasites followed a different trajectory. At H24, the samples were more similar to the H0 profile than the untreated H24 parasites indicating a delay in the normal transcriptional pathway.
- Differential gene expression analysis identified substantial transcriptional changes following GBPS336 exposure in ring-stage cultures. The number of differentially expressed genes was highest at H24 and decreased by H48, indicating that the transcriptional response was most pronounced during the early phase of drug exposure. By contrast, no gene reached statistical significance in trophozoite-stage cultures after exclusion of cycle-related genes. The absence of statistically significant differentially expressed genes in trophozoite-stage cultures should be interpreted cautiously. Two trophozoite-stage libraries were excluded because of insufficient sequencing depth, and these cultures also showed lower parasitaemia, both of which may have limited the statistical power to detect transcriptional differences at this stage.
- Accordingly, subsequent differential expression and enrichment analyses (Section 3.3 and Section 3.4) refer specifically to the ring-stage comparisons. Hierarchical clustering showed coherent transcriptomic profiles among biological replicates and distinct patterns between treated and untreated parasites (Figure 7).

### 3.3. Functional Enrichment Analysis Identifies Coordinated Biological Programs Affected by GBPS336

- To characterize the biological program associated with the transcriptional response to GBPS336, differentially expressed genes were subjected to a GO enrichment analysis (Figure 8). Enriched GO categories were identified at both H24 and H48, indicating that the differentially expressed genes were not randomly distributed across functional categories.
- At H24, three GO categories showed significant enrichment. The GO:0009986 category “cell surface” included 26 differentially expressed genes out of the 156 genes in the category (adjusted *p* = 0.011), while the GO:0070258 category “inner membrane pellicle complex” had 15 out of 66 (adjusted *p* = 0.013), and the GO category GO:0044228 “host cell surface” had 10 out of 31 (adjusted *p* = 0.032). The genes contributing to these enriched categories are detailed in Supplementary Tables S1–S3. At H48, only the GO category GO:0009986 “cell surface” remained significantly enriched, with 17 differentially expressed genes out of 156 (adjusted *p* = 4.03 × 10^−6^), the corresponding differentially expressed genes are provided in Supplementary Table S4.
- The most highly enriched GO categories were associated with the parasite’s structural organization and host–cell interactions. These included the parasite surface, host–cell surface, the inner membrane complex, the pellicle and other membrane-associated compartments. Several categories related to erythrocyte invasion and parasite morphogenesis were also systematically enriched. These findings identify invasion-related functions among the transcriptional program perturbed by GBPS336, providing mechanistic insights into the parasite’s response to drug exposure and identifying potential pathways for further investigation. Although the number of significantly enriched GO categories decreased at H48 compared with H24, the persistent enrichment of the “parasite cell-surface” category at both time points highlights the sustained transcriptional modulation of processes involved in parasite surface organization during GBPS336 exposure.

### 3.4. Representative Gene Families Support the Hypothesis of Coordinated Remodelling of the Parasite–Host Interface

Representative gene families provide illustrative examples of the biological programs identified by GO enrichment analysis which are systematically associated with the parasite’s structural organization, membrane compartments and parasite–host interactions:

- Among the downregulated functional modules, genes encoding proteins associated with the glideosome and the inner membrane complex consistently showed reduced expression together with proteins involved in membrane complex organization. Downregulation was also observed for several genes encoding merozoite surface- and invasion-associated proteins, including members of the 6-cysteine protein family (P38 and P41) (Supplementary Tables S1 and S2).
- Conversely, several members of the *SURFIN* multigene family (*SURFIN1.1*, *SURFIN1.3*, *SURFIN8.1*, *SURFIN8.3* and *SURFIN14.1*) exhibited upregulation following GBPS336 exposure. A similar transcriptional profile was observed for several PHIST family members and other membrane-associated and exported proteins associated with the parasite–host interface (Supplementary Tables S1, S3 and S4).

## 4. Discussion

In this study, a combined RNA-Seq and phenotype analysis was employed to elucidate the effects of arylaminoalcohol derivative GBPS336 on *P. falciparum*. Our results indicate that GBPS336 disturbs the normal development of *P. falciparum*. This effect, first evidenced by microscopic examination, is supported by changes in the transcription programs involved in parasite organization and interactions with the host cell.

Two approaches converge on the same conclusion. Microscopic analysis evidenced a rapid arrest of intraerythrocytic development, characterized by vacuolization and pyknosis. Similarly, RNA sequencing showed that the transcriptional profile of GBPS336-treated ring-stage parasites at H24 remained more similar to that observed at H0 than to the transcriptional profile expected during normal parasite development. This finding suggests that GBPS336 exposure delays the normal transcriptional progression associated with parasite maturation. Parasite development during the blood stage is controlled by a tightly regulated transcriptional program that can be altered by both genetic and chemical treatments [21,22,23].

A noticeable result of this study was the preservation of coherent transcriptional profiles despite the marked morphological alterations induced by GBPS336. Treated parasites displayed consistent gene expression patterns across biological replicates, indicating that the transcriptional response remained structured despite the disruption of normal parasite development. These findings suggest that GBPS336 alters the transcriptional program associated with parasite development rather than causing a generalized loss of transcriptional organization.

Within this structured transcriptional response, the enrichment of genes associated with erythrocyte invasion and the inner membrane complex is of particular interest. Importantly, these findings should not be interpreted as evidence that GBPS336 directly targets a specific invasion-related protein or receptor. These transcriptional changes may reflect either a direct molecular response to the compound or a secondary consequence of altered parasite development, cellular stress, or growth deceleration. Functional studies will therefore be required to determine whether these pathways are directly involved in the biological response to GBPS336 or represent downstream consequences of its antiplasmodial activity.

The representative gene families identified in this study provide illustrative examples of this functional remodeling. Components of the inner membrane complex and glideosome were predominantly downregulated, whereas several *SURFIN* and *PHIST* genes showed parallel upregulation. Although most of these proteins remain incompletely characterized, they are generally associated with parasite structure, motility, and interactions with erythrocytes [24,25]. These expression changes therefore provide candidate pathways for further investigation rather than evidence of a direct molecular target of GBPS336.

Our study has several limitations, first, two trophozoite-stage libraries were excluded because of insufficient sequencing data. In addition, the lower parasitaemia observed in trophozoite-stage cultures may have further limited the power to detect transcriptional changes at this stage. Consequently, the transcriptomic response to GBPS336 could not be robustly characterized at the trophozoite stage. Repeating RNA-seq experiments with adequate sequencing depth at this developmental stage would therefore be valuable to determine whether the transcriptional alterations observed at the ring stage are maintained, evolve, or are replaced by stage-specific responses during parasite development. Second, the exclusion of 2452 genes associated with developmental-stage differences reduced confounding bias from the parasite cell cycle but may also have removed genes that are both stage-dependent and modulated by GBPS336. Third, no reference antimalarial treatment was used as a pharmacological control. Inclusion of a standard antimalarial agent, such as chloroquine or an artemisinin derivative, in parallel experiments could have provided a comparative framework for assessing the phenotypic and transcriptional responses induced by GBPS336. Such comparisons would be useful for determining whether the alterations observed with GBPS336 overlap with or differ from those induced by established antimalarial drugs. Fourth, there is no independent validation of selected transcriptomic changes by RT-qPCR. Although the RNA-seq data provide a global overview of the transcriptional response to GBPS336, targeted validation of representative differentially expressed genes, particularly those associated with the enriched GO categories, will be considered in future studies to further support and characterize these findings.

Based on the present findings, further studies are warranted to better characterize the biological response to GBPS336. In particular, independent validation of selected transcriptomic changes, complementary molecular approaches, and experiments covering additional intraerythrocytic developmental stages will be important to explore. Evaluation against additional *P. falciparum* strains and in combination with established antimalarial drugs would further define its potential for preclinical development.

## 5. Conclusions

Overall, our findings demonstrate that GBPS336 significantly affects the intraerythrocytic development of *P. falciparum* and is associated with marked transcriptional and morphological changes. The transcriptomic response highlights parasite surface, inner membrane complex and parasite–host interaction pathways as potential components of the response to treatment. These findings provide a basis for further investigation of the biological mechanisms underlying the antimalarial activity of GBPS336.

## Figures and Tables

**Figure 5:**
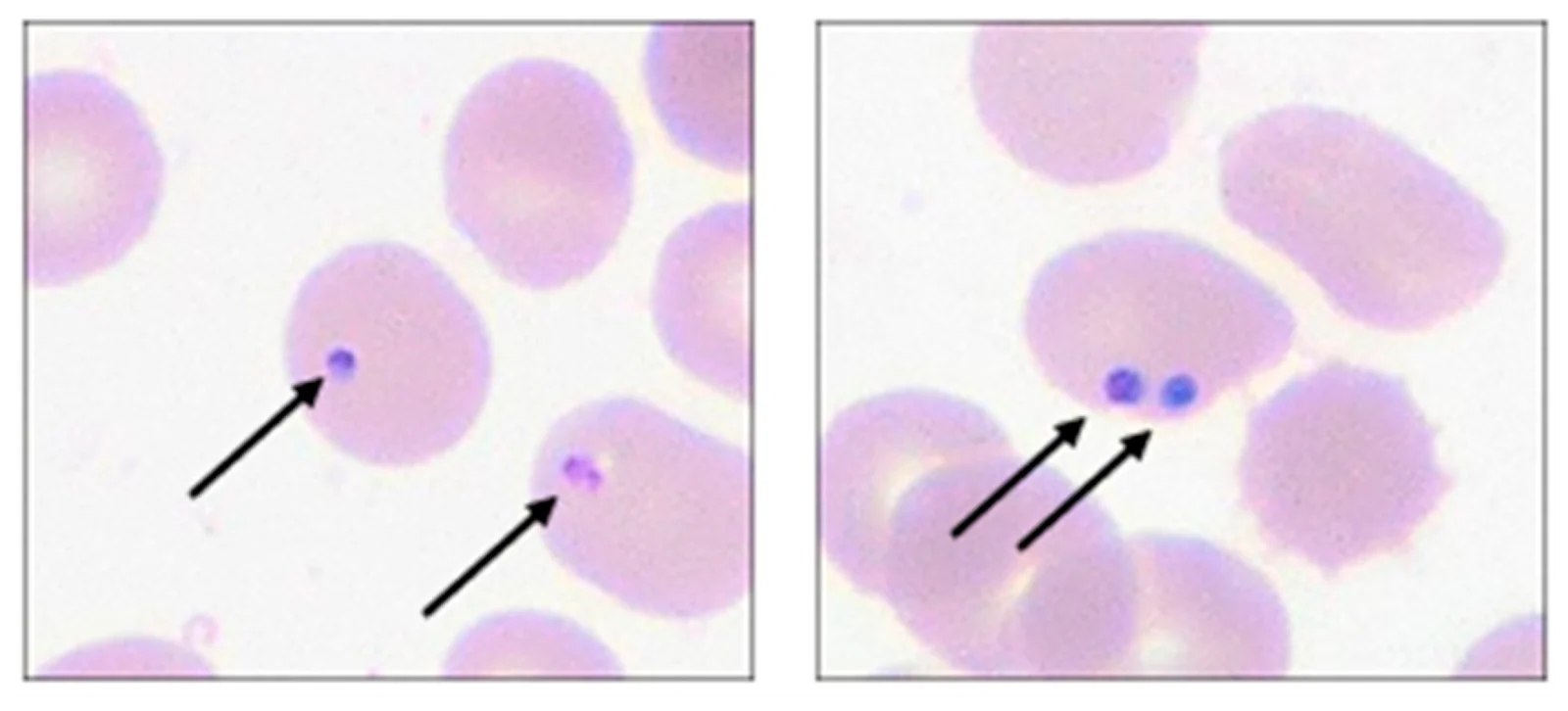
Pyknotic forms (arrows) from GBPS336-treated cultures (thin blood smears, May–Grünwald–Giemsa staining, ×1000).

**Figure 6:**
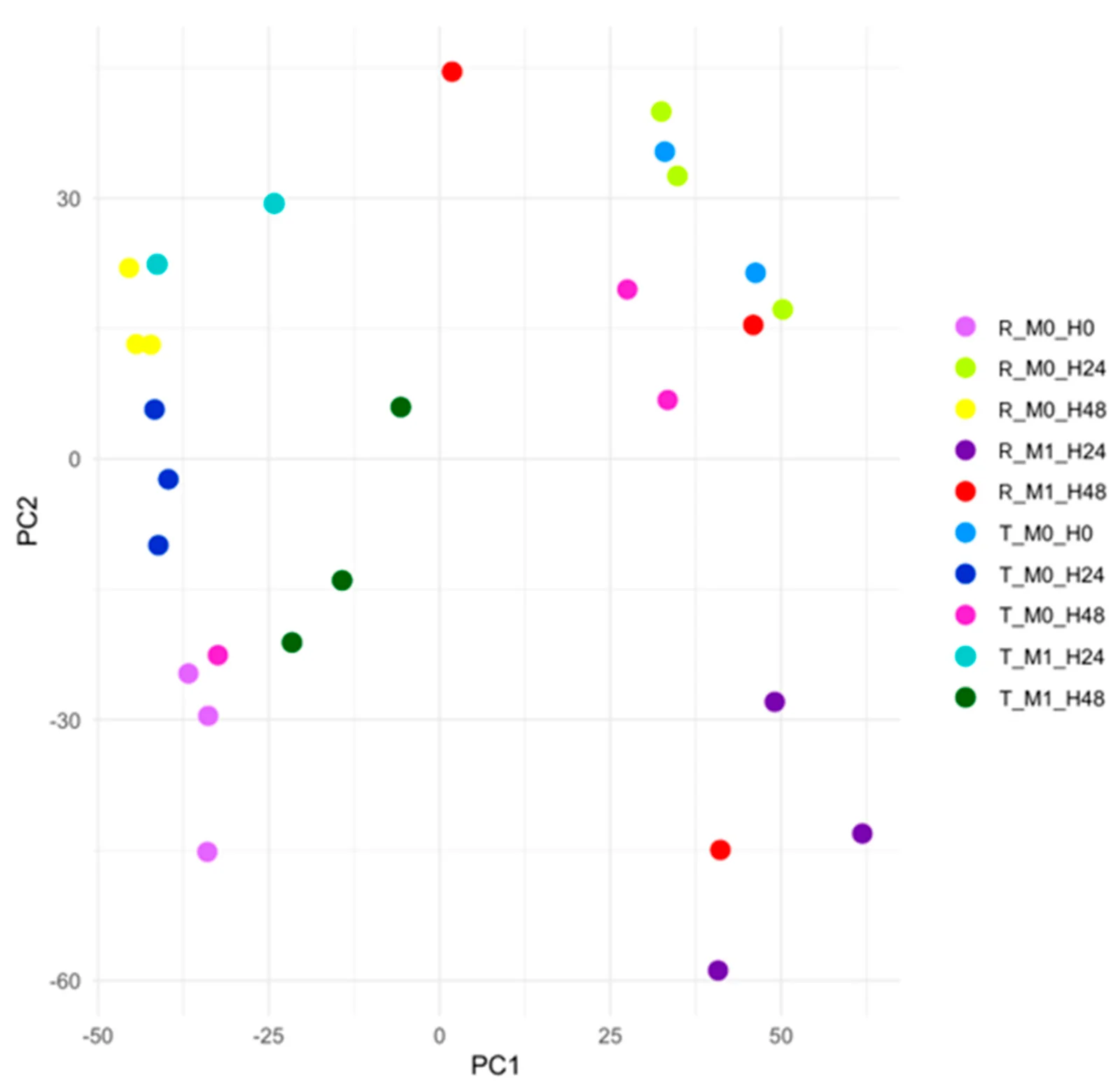
Principal component analysis (PCA) of transcriptomic profiles from cultures synchronized at the ring and trophozoite stages at H0, H24 and H48. R, ring stage; T, trophozoite stage; M0, control; M1, GBPS336-treated. Each color corresponds to one condition and each point to one replicate. PC1 and PC2, first and second principal components; the percentage of explained variance is presented on each axis.

**Figure 7:**
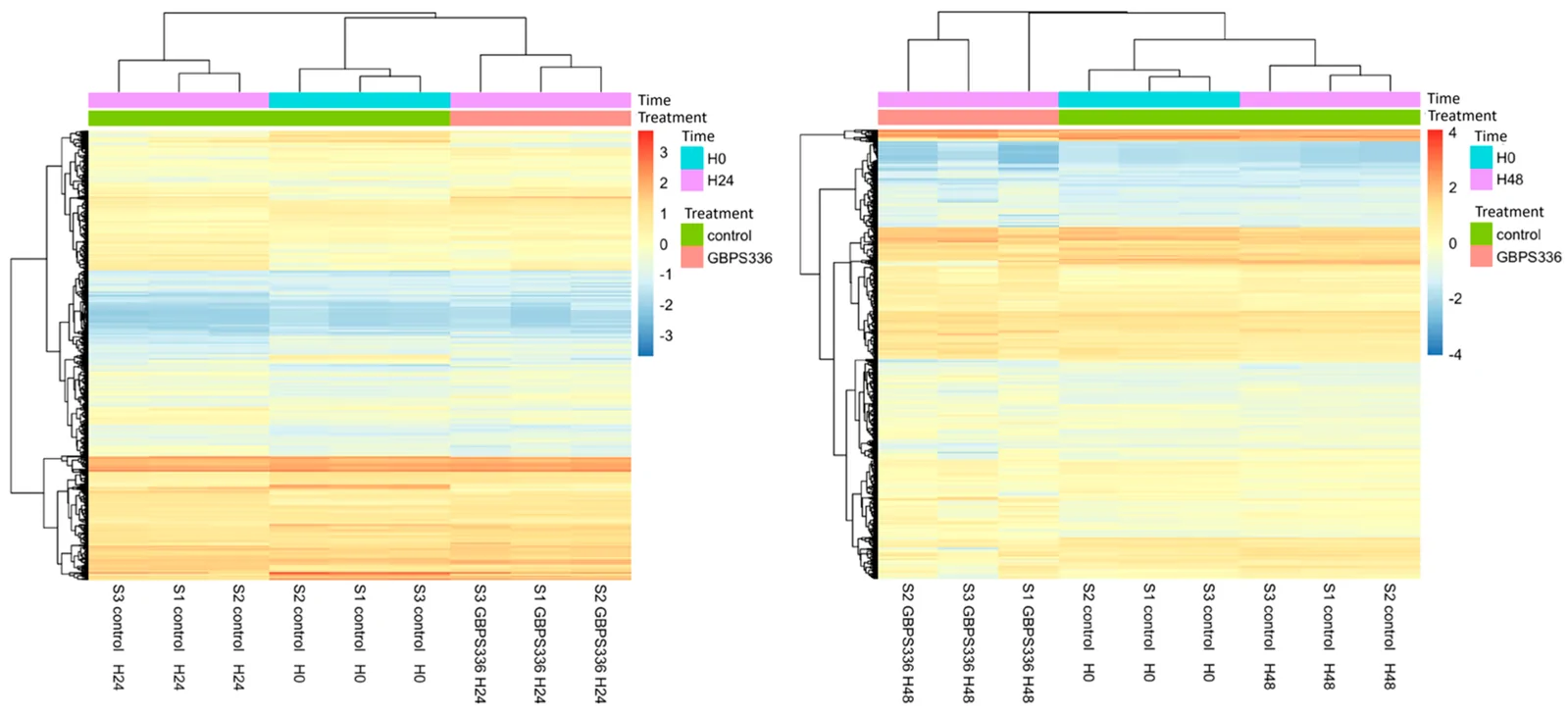
Heatmaps from hierarchical clustering analysis of gene expression in cultures synchronized at the ring stage. Left: comparison of the transcriptomic profiles of cultures at H0 and H24, with and without GBPS336. Right: comparison of the transcriptomic profiles of cultures at H0 and H48, with and without GBPS336. S1, S2 and S3 denote the replicates.

**Figure 8:**
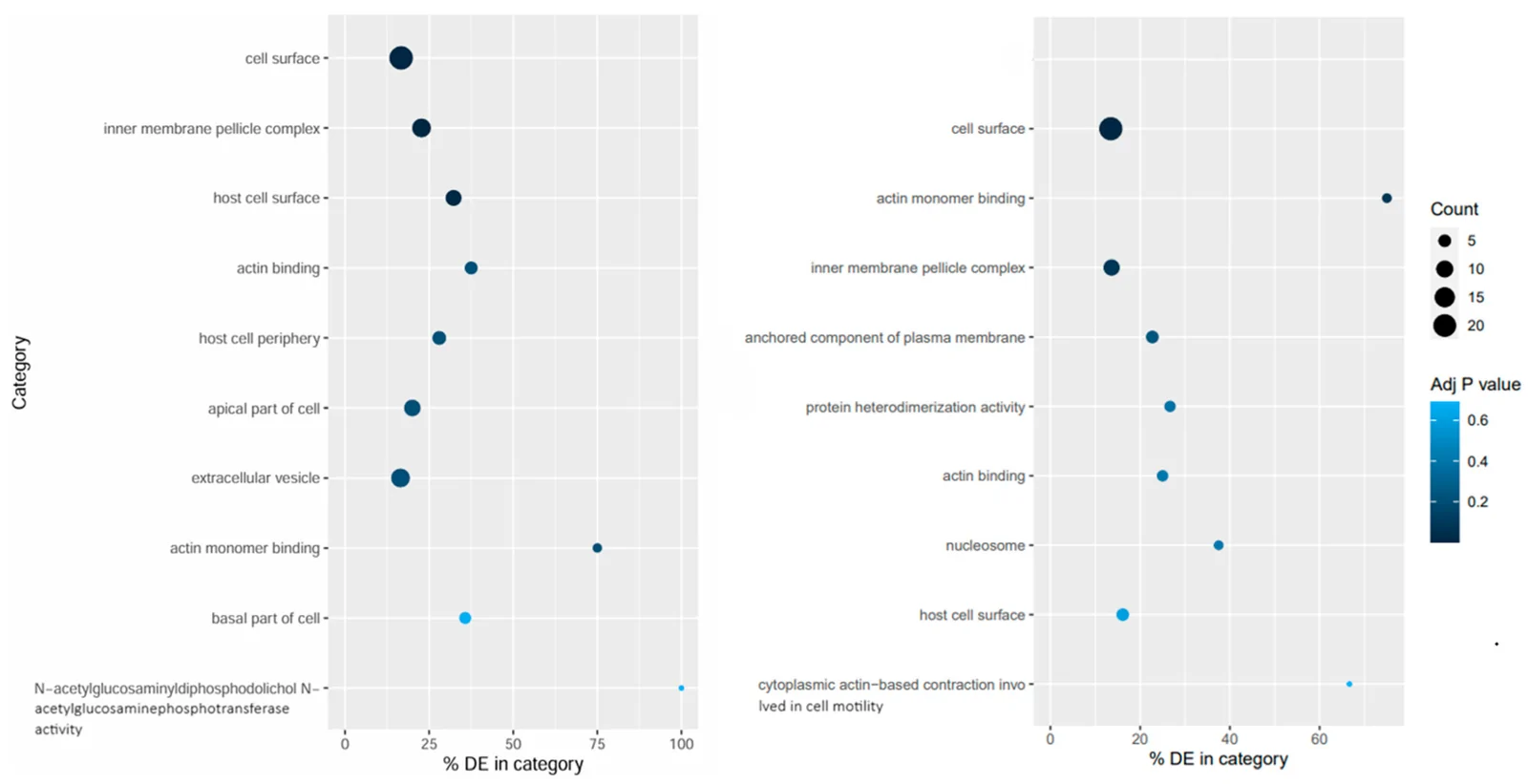
Enrichment analysis showing the top GO categories with the greatest changes in expression in cultures synchronized at the ring stage (Wallenius method). (**Left**) comparison of cultures with and without GBPS336 at H24. (**Right**) comparison of cultures with and without GBPS336 at H48. The horizontal axis (%DE in category) indicates the percentage of differentially expressed genes in each category. Point size reflects the total number of genes in the category. Colour intensity indicates the adjusted *p*-value (Adj *p*-value).

## Data Availability

The original contributions presented in this study are included in the article/Supplementary Material. Further inquiries can be directed to the corresponding author.

## Supplementary Materials

The following supporting information can be downloaded at https://www.mdpi.com/article/10.3390/biology15201837/s1: Table S1: Genes in the GO category “cell surface” (GO:0009986) showing significant differential expression between ring-stage-synchronized cultures with and without GBPS336 at H24; Table S2: Genes in the GO category “inner membrane pellicle complex” (GO:0070258) showing significant differential expression between ring-stage-synchronized cultures with and without GBPS336 at H24; Table S3: Genes in the GO category “host cell surface” (GO:0044228) showing significant differential expression between ring-stage-synchronized cultures with and without GBPS336 at H24; Table S4: Genes in the GO category “cell surface” (GO:0009986) showing significant differential expression between ring-stage-synchronized cultures with and without GBPS336 at H48.

## Abbreviations

The following abbreviations are used in this manuscript:

ACT	Artemisinin-based Combination Therapy
cDNA	complementary DNA
DEGs	Differentially Expressed Genes
dUTP	deoxyuridine triphosphate
GO	Gene Ontology
H0	baseline (time 0)
H24	24 h after drug exposure
H48	48 h after drug exposure
IC50	Half maximal Inhibitory Concentration
log_2_FC	log_2_ fold change
M0	untreated control
M1	GBPS336-treated condition
*P. falciparum*	*Plasmodium falciparum*
PC1	first principal component
PC2	second principal component
PCA	Principal Component Analysis
PCR	Polymerase Chain Reaction
PfEMP1	*P. falciparum* Erythrocyte Membrane Protein 1
PHIST	Plasmodium Helical Interspersed Subtelomeric protein
R	ring-stage
RNA	Ribonucleic Acid
RNA-seq	RNA sequencing
RPMI	Roswell Park Memorial Institute
RT-qPCR	Reverse Transcription quantitative Polymerase Chain Reaction
SURFIN	Surface-associated Interspersed protein
T	trophozoite stage
