# Supplementary material for: Integrated Phenotypic and Transcriptomic Analysis of the Effects of a Novel Arylaminoalcohol on Plasmodium falciparum
Source: Biology (Basel). 2026 Oct 14;15(20):1837. doi: 10.3390/biology15201837 (PMC13701406; doi:10.3390/biology15201837)
Supplement: Supplementary file 1 [file biology-15-01837-s001.zip › biology-4608156-supplementary.pdf]

Supplementary Materials

Table S1. Genes in the GO category "cell surface" (GO:0009986) showing significant differential expression between ring-stage-synchronized cultures with and without GBPS336 at H24.

Gene ID	Log_2FC	*P* value adj	Product
PF3D7_0104100	-3.37	1.02E-06	conserved *Plasmodium* membrane protein, unknown function
PF3D7_0113100	1.42	1.73E-04	surface-associated interspersed protein 1.1 (SURFIN 1.1)
PF3D7_0115000	1.73	7.04E-03	surface-associated interspersed protein 1.3 (SURFIN 1.3)
PF3D7_0208900	1.84	3.67E-06	6-cysteine protein P230p
PF3D7_0404900	-2.62	2.18E-11	6-cysteine protein P41
PF3D7_0423500	-2.31	5.87E-10	glideosome associated protein with multiple membrane spans 2
PF3D7_0506900	-2.78	9.40E-09	rhomboid protease ROM4
PF3D7_0508000	-4.88	7.16E-24	6-cysteine protein P38
PF3D7_0511400	1.60	1.27E-04	conserved *Plasmodium* protein, unknown function
PF3D7_0515700	-3.03	2.60E-11	glideosome-associated protein 40, putative
PF3D7_0800700	1.94	4.89E-03	surface-associated interspersed protein 8.3 (SURFIN 8.3)
PF3D7_0818900	1.11	1.06E-03	heat shock protein 70
PF3D7_0831100	1.91	2.58E-05	surface-associated interspersed protein 8.1 (SURFIN 8.1)
PF3D7_0905400	-3.80	5.20E-11	high molecular weight rhoptry protein 3
PF3D7_0909500	-1.51	1.89E-02	subpellicular microtubule protein 1, putative
PF3D7_1132800	-1.45	9.44E-08	aquaglyceroporin
PF3D7_1140400	-2.92	5.12E-03	conserved *Plasmodium* protein, unknown function
PF3D7_1228600	-3.77	1.46E-10	acidic basic repeat antigen
PF3D7_1238000	-1.23	3.90E-02	COPI associated protein, putative
PF3D7_1252100	-3.68	2.85E-10	rhoptry neck protein 3
PF3D7_1323700	-3.34	6.04E-11	glideosome associated protein with multiple membrane spans 1
PF3D7_1406800	-3.06	4.26E-13	glideosome associated protein with multiple membrane spans 3
PF3D7_1409400	-1.99	1.02E-09	conserved protein, unknown function
PF3D7_1431900	-2.40	1.23E-07	inner membrane complex suture component, putative
PF3D7_1437300	-2.50	4.62E-07	conserved *Plasmodium* protein, unknown function
PF3D7_1477600	2.19	3.78E-04	surface-associated interspersed protein 14.1 (SURFIN 14.1)

Table S2. Genes in the GO category "inner membrane pellicle complex" (GO:0070258) showing significant differential expression between ring-stage-synchronized cultures with and without GBPS336 at H24.

Gene ID	Log_2FC	*P* value adj	Product
PF3D7_0423500	-2.31	5.87E-10	glideosome associated protein with multiple membrane spans 2
PF3D7_0510300	1.52	4.66E-03	stripes inner membrane complex protein, putative
PF3D7_0515700	-3.03	2.60E-11	glideosome-associated protein 40, putative
PF3D7_0522600	-2.66	4.74E-05	inner membrane complex protein
PF3D7_0609800	-1.95	5.69E-05	palmitoyltransferase DHHC2, putative
PF3D7_0611600	-3.27	2.61E-12	basal complex transmembrane protein 1
PF3D7_0702500	1.64	3.66E-06	*Plasmodium* exported protein, unknown function
PF3D7_0704100	-2.26	1.64E-08	basal complex transmembrane protein 2
PF3D7_0722900	-2.33	8.60E-05	conserved protein, unknown function
PF3D7_1205200	-3.49	1.32E-02	HAD domain ookinete protein, putative
PF3D7_1229300	-2.88	2.12E-10	conserved *Plasmodium* protein, unknown function
PF3D7_1323700	-3.34	6.04E-11	glideosome associated protein with multiple membrane spans 1

PF3D7_1355600	1.27	3.68E-03	PhIL1 interacting protein PIP1
PF3D7_1406800	-3.06	4.26E-13	glideosome associated protein with multiple membrane spans 3
PF3D7_1431900	-2.40	1.23E-07	inner membrane complex suture component, putative

Table S3. Genes in the GO category "host cell surface" (GO:0044228) showing significant differential expression between ring-stage-synchronized cultures with and without GBPS336 at H24.

Gene ID	Log_2FC	*P* value adj	Product
PF3D7_0113000	1.29	8.60E-05	glutamic acid-rich protein GARP
PF3D7_0113100	1.42	1.73E-04	surface-associated interspersed protein 1.1 (SURFIN 1.1)
PF3D7_0115000	1.73	7.04E-03	surface-associated interspersed protein 1.3 (SURFIN 1.3)
PF3D7_0201600	1.32	7.85E-06	PHISTb domain-containing RESA-like protein 1
PF3D7_0401800	2.29	2.56E-05	*Plasmodium* exported protein (PHISTb), unknown function
PF3D7_0532400	1.11	3.01E-04	lysine-rich membrane-associated PHISTb protein
PF3D7_0800700	1.94	4.89E-03	surface-associated interspersed protein 8.3 (SURFIN 8.3)
PF3D7_0831100	1.91	2.58E-05	surface-associated interspersed protein 8.1 (SURFIN 8.1)
PF3D7_1249000	1.88	2.61E-08	conserved *Plasmodium* membrane protein, unknown function
PF3D7_1477600	2.19	3.77E-04	surface-associated interspersed protein 14.1 (SURFIN 14.1)

Table S4. Genes in the GO category "cell surface" (GO:0009986) showing significant differential expression between ring-stage-synchronized cultures with and without GBPS336 at H48.

Gene ID	Log_2FC	*P* value adj	Product
PF3D7_0102700	-3.83	5.26E-19	merozoite-associated tryptophan-rich antigen
PF3D7_0113100	1.93	3.58E-03	Surface-associated interspersed protein 1.1 (SURFIN 1.1)
PF3D7_0501200	1.73	3.57E-03	Parasite-infected erythrocyte surface protein
PF3D7_0629300	-3.53	2.21E-07	Phospholipase, putative
PF3D7_0702300	-2.73	1.71E-04	sporozoite threonine and asparagine-rich protein
PF3D7_0903700	-1.11	2.53E-01	alpha tubulin 1
PF3D7_1035300	-2.97	2.96E-04	Glutamate-rich protein GLURP
PF3D7_1035400	-3.34	8.31E-09	merozoite surface protein 3
PF3D7_1035500	-3.59	1.03E-06	merozoite surface protein 6
PF3D7_1036000	-2.96	7.03E-08	merozoite surface protein 11
PF3D7_1218000	-3.18	3.34E-07	thrombospondin-related apical membrane protein
PF3D7_1228600	-2.47	1.41E-02	acidic basic repeat antigen
PF3D7_1420700	-1.25	3.43E-02	Surface protein P113

Differential gene expression was considered significant at $log_2FC > 1$ and adjusted $p < 0.05$.
